# Impact of circulating lymphoma cells at diagnosis on outcomes in patients with newly diagnosed *de novo* diffuse large B-cell lymphoma

**DOI:** 10.1186/s13045-024-01658-y

**Published:** 2025-01-05

**Authors:** Sayan Mullick Chowdhury, Subodh Bhatta, Timothy J. Voorhees, Kaitlin Annunzio, David A. Bond, Yazeed Sawalha, Audrey Sigmund, Walter Hanel, Lalit Sehgal, Lapo Alinari, Robert Baiocchi, Kami Maddocks, Beth Christian, Dan Jones, Narendranath Epperla

**Affiliations:** 1 Division of Hematology, Arthur G. James Cancer Hospital and Richard J. Solove Research Institute, The Ohio State University, Columbus, OH USA; 2 Department of Pathology, The Ohio State University, Columbus, OH USA; 3 Division of Hematology and Hematologic Malignancies, Huntsman Cancer Institute, University of Utah, Salt Lake City, UT USA; 4 Division of Hematology and Hematologic Malignancies, Department of Medicine, Huntsman Cancer Institute, Salt Lake City, UT 84103 USA

**Keywords:** Diffuse large B-cell lymphoma, DLBCL, Circulating lymphoma cells, CL, Prognosis, Progression-free survival, Overall survival

## Abstract

Diffuse large B-cell lymphoma (DLBCL), the most common B-cell non-Hodgkin lymphoma rarely presents with circulating lymphoma cells (CL) at diagnosis. Previous studies were limited by small sample size precluding robust analysis. Hence, we evaluated the prognostic relevance of CL cells in newly diagnosed DLBCL patients. Based on peripheral blood (PB) immunophenotyping, patients were grouped into CL + and CL−. CL was defined as detectable clonally restricted B-cells that matched the actual or expected B-cell immunophenotype of DLBCL. The primary endpoint was progression-free survival (PFS), and secondary endpoints included overall survival (OS) and diagnosis-to-treatment interval (DTI). Among the 1266 patients with DLBCL, 621 had PB flow at diagnosis, and after excluding patients not meeting eligibility criteria, 588 cases remained. Among these, 85 (15%) were CL + and 503 were CL- (85%). Patients in CL + group were younger (67 vs. 70 years, *p* = 0.03) with a higher proportion of non-bulky disease (85% vs. 56%, *p* < 0.0001), normal albumin (79% vs. 54%, *p* < 0.0001), and *MYC/BCL2* and/or *BCL6* rearrangements (18% vs. 7%, *p* = 0.003) compared to the CL − group. Patients with CL at diagnosis had significantly inferior PFS and OS compared with those without CL. After adjusting for factors associated with inferior PFS and OS in univariable analysis, presence of CL remained significantly associated with inferior PFS (HR = 2.04, 95%CI = 1.47–2.84, *p* < 0.0001) and OS (HR = 1.61, 95%CI = 1.1–2.36, *p* = 0.01), respectively. There was no significant difference in DTI between the two groups. Given the prognostic relevance associated with presence of CL, clinicians should consider checking PB flow at diagnosis in all newly diagnosed DLBCL patients.

**Supplementary Information:**

The online version contains supplementary material available at 10.1186/s13045-024-01658-y.

**To the editor,**

Diffuse large B-cell lymphoma (DLBCL) is the most common non-Hodgkin lymphoma and accounts for ~ 30–40% of all lymphoid malignancies [1, 2]. Although patients with advanced stage DLBCL have frequent bone marrow involvement, circulating lymphoma cells (CL) in peripheral blood (PB) is a relatively rare finding, with only a few studies having reported/evaluated this previously [3–8]. Therefore, we performed a retrospective study of adults with *de novo* DLBCL (diagnosed in or after 2010) to evaluate the prognostic impact of CL at diagnosis.

To be eligible, patients must have had PB immunophenotyping via flow cytometry at diagnosis and received anthracycline-based chemotherapy in first-line setting. Patients with transformed indolent lymphomas, Richter’s transformation, and PCNSL were excluded. CL was defined as detectable kappa or lambda-restricted B-cells that matched the actual or expected B-cell immunophenotype of DLBCL. Flow findings were correlated with hematopathologist morphologic review of the peripheral smear. Eligible patients were divided into CL + and CL- groups based on the presence or absence of CL at diagnosis. The primary endpoint was progression-free survival (PFS), while secondary endpoints included comparing overall survival (OS) and diagnosis-to-treatment interval (DTI) between the two groups. Definitions and statistical analysis are detailed in the Supplementary appendix.

Among 1266 patients with newly diagnosed DLBCL, 621 had PB flow at diagnosis. After excluding patients not meeting the eligibility criteria, 588 cases remained (Figure S1) with 85 (14.5%) CL + cases. Table 1 shows the baseline characteristics of DLBCL patients categorized by the presence or absence of CL. Details pertaining to the first-line treatment regimens are outlined in Table S1. There was no significant difference in the receipt of first-line therapies between CL + and CL- groups (*p* = 0.26, Table S2).

**Table 1:** Baseline characteristics

Variable	All *N* = 588 (%)	CL- *n* = 503 (%)	CL+ *n* = 85 (%)	*p*-value
Median age, years (range)	70 (22–90)	70 (22–90)	67 (39–97)	**0.03**
Gender				0.13
Male	305 (52)	254 (51)	51 (60)	
Female	283 (48)	249 (49)	34 (40)	
Race				0.40
White	552 (94)	470 (93)	82 (97)	
Other	36 (6)	33 (7)	3 (3)	
ECOG PS				0.07
0–1	350 (60)	292 (59)	58 (70)	
2–3	232 (40)	207 (41)	25 (30)	
Stage				**< 0.001**
1–2	49 (8)	49 (10)	0	
3–4	537 (92)	452 (90)	85 (100)	
B-symptoms				0.74
No	265 (51)	221 (50)	44 (53)	
Yes	257 (49)	218 (50)	39 (47)	
Bulky Disease				**< 0.001**
No	353 (61)	282 (56)	71 (85)	
Yes	231 (39)	219 (43)	12 (15)	
Low albumin				**< 0.001**
No	338 (58)	272 (54)	66 (79)	
Yes	248 (42)	230 (46)	18 (21)	
LDH > ULN				0.14
No	152 (26)	124 (24)	28 (33)	
Yes	436 (74)	379 (75)	57 (67)	
Cell of origin				0.09
GCB	234 (40)	205 (41)	29 (34)	
Non-GCB	195 (33)	158 (31)	37 (44)	
Unknown	159 (27)	140 (28)	19 (22)	
*MYC* rearrangement				**0.002**
No	456 (86)	400 (88)	56 (74)	
Yes	74 (14)	54 (12)	20 (26)	
DHL/THL				**0.003**
No	485 (91)	422 (93)	63 (82)	
Yes	46 (9)	32 (7)	14 (18)	
R-IPI Prognostic score				0.15
0	17 (3)	17 (3)	0	
1–2	463 (79)	391 (78)	72 (85)	
3–5	108 (18)	95 (19)	13 (15)	
Median f/up time among survivors from the start of treatment, years (range)	5.47 (0.02–21.56)	5.48 (0.02–21.56)	5.37 (0.7–13.40)	**0.048**

Abbreviations: CL-, circulating lymphoma cells absent; CL+, circulating lymphoma cells present; LDH, lactate dehydrogenase; ULN, upper limit of normal, GCB-Germinal Center B Cell like, DHL -Double Hit Lymphoma, THL-Triple Hit Lymphoma, R-IPI, Revised international prognostic index

Overall response rate (ORR) and complete response rate (CRR) to first-line therapy in the entire cohort were 86% and 73%, respectively. When evaluated by CL, CL + group had a significantly inferior ORR (78% vs. 87%, *p* = 0.02) and CRR (59% vs. 75%, *p* < 0.01) compared to CL- group (Table S3).

Median PFS and OS in the entire cohort was 7.35 years (95%CI = 6.18–8.49) and 9.8 years (95%CI = 9.1–10.4), respectively. Median PFS (1.7 versus 7.93 years, *p* < 0.0001, Fig. 1A) and OS (7.82 versus 10.04 years, *p* = 0.0019, Fig. 1B) were significantly inferior in the CL + group compared to CL- group. After adjusting for factors associated with inferior PFS and OS in univariable analysis (CL+, bulky disease, and DHL/THL), CL + remained associated with significantly inferior PFS (HR = 2.04, 95%CI = 1.47–2.84, *p* < 0.001, Table S4) and OS (HR = 1.61, 95%CI = 1.1–2.36, *p* = 0.01, Table S5) compared to CL- cohort in the multivariable analysis.

**Fig. 1:**
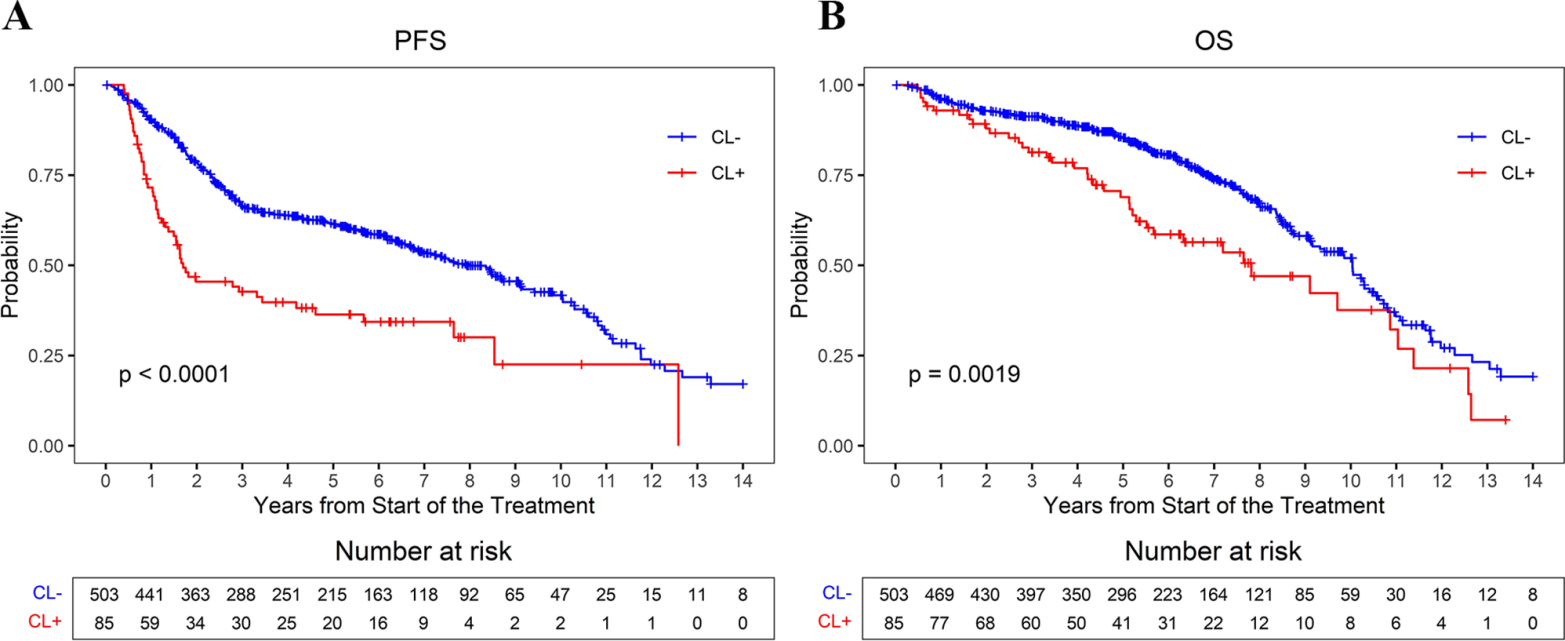
Progression-free survival and overall survival in patients with newly diagnosed DLCBL based on the presence (CL+) or absence (CL-) of CL cells. (**A**) PFS, (**B**) OS

Median DTI was not significantly different between the CL+ (0.92 months, 95%CI = 0.69–1.25) versus CL- group (0.99 months, 95%CI = 0.92–1.08, *p* = 0.50).

On analyzing outcomes based on the type of first-line therapy, we found that median PFS was significantly inferior in the CL + compared to CL- cohort regardless of the type of first-line therapy (Figures S2A and S2B). While median OS was significantly inferior among those who received first-line intensive-induction chemotherapies (IIC) in the CL + versus CL- group (Figure S3A), it was not statistically different between the two groups among R-CHOP recepients (Figure S3B). Given this, we performed a sensitivity analysis for OS among the recipients of IIC alone (controlling for other high-risk factors such as DHL/THL) and found that presence of CL was independently associated with inferior OS in the Cox model (HR = 2.32, 95%CI = 1.4–3.84, *p* = 0.001).

As all patients in CL + group are stage 4, we performed additional analysis by dividing patients into 3 groups, stage 1–3 CL-, stage 4 CL-, and CL+ (Table S6). The response rates (Table S7) and survival outcomes (Figures S4A and S4B) in the 3 groups were in line with the main analysis.

Limitations include a nonuniform selection of patients with *de novo* DLBCL for the performance of PB flow at diagnosis and likley overestimation of incidence of CL + due to the requirement of flow for eligibility.

In this largest study-to-date evaluating the impact of CL on outcomes in patients with newly diagnosed *de novo* DLBCL, we found that presence of CL at diagnosis was associated with inferior response rates and survival compared to those without CL. Given the prognostic relevance associated with the presence of CL, clinicians should consider checking PB flow at diagnosis in all newly diagnosed patients with DLBCL.

## Electronic supplementary material

Below is the link to the electronic supplementary material.

Supplementary Material 1

## Data Availability

Data is available upon request to the corresponding author as permitted by the IRB.
