# Supplementary material for: Impact of circulating lymphoma cells at diagnosis on outcomes in patients with newly diagnosed de novo diffuse large B-cell lymphoma
Source: J Hematol Oncol. 2025 Jan 5;18:4. doi: 10.1186/s13045-024-01658-y (PMC11702192; doi:10.1186/s13045-024-01658-y)

**SUPPLEMENTAL APPENDIX**

**Table of Contents** 1

**Patients and Methods**  2

**Breakdown of intensive induction therapies:** Table S1 3

**Treatments received and overall response:** Table S2 4

**Response rates after first line treatment in CL+ vs CL- groups:** Table S3 5

**PFS Modeling (Univariable and Multivariable analysis):** Table S4 6

**OS Modeling (Univariable and Multivariable analysis):** Table S5 8

**Baseline characteristics based on stage 1-3 and 4 CL- and CL+:** Table S6 10

**Response rates based on stage 1-3 and 4 CL- and CL+ cohorts:** Table S7 11

**Consort Diagram:** Figure S1 12

**PFS between CL+ and CL- groups:** Figure S2 13

**OS between CL+ and CL- groups:** Figure S3 14

**PFS and OS between stage 1-3 and 4 CL- and CL+ groups:** Figure S4 15

**Patients and Methods**

**Definitions**

PFS was defined as the time from initiation of first-line therapy to relapse, progression, or death from any cause, censoring those alive without relapse or progression at the last contact. OS was calculated from the start of first-line therapy to death from any cause or censoring those alive at the last follow-up. DTI was defined as the time from diagnosis to initiation of first-line systemic therapy.

**Variables of interest**

Additional data, such as demographic and clinical patient information, were obtained from the electronic medical record. Laboratory values known to be associated with DLBCL prognosis were also collected, including age, ECOG (Eastern Cooperative Oncology Group) performance status stage, lactate dehydrogenase (LDH), albumin, presence of bulky disease, cell of origin (COO), MYC/BCL2 and or BCL6 rearrangement status. Treatment data, including first-line therapy, response to first-line therapy, and date of relapse, progression, or death, were also collected.

**Statistical Analysis**

Demographic and disease characteristics were described using descriptive statistics. Continuous variables were summarized with their median and range and compared between the groups using the Wilcoxon signed rank test or Kruskal-Wallis Test. Categorical variables were presented with their frequency and percentage and compared between the groups using the Chi-square test or Fisher's exact test, depending on the data type. The Kaplan-Meier method was used to estimate the survival outcomes, and the groups were compared using the log-rank test. The hazard ratios associated with the risk of progression or death were estimated using Cox proportional hazard regression models. The multivariable analysis for OS and PFS incorporated significant variables from the univariable analysis and clinically essential variables. The proportional hazard assumption was checked using the Goodness-of-fit test after fitting Cox models. Analyses were performed using R version 4.3.2, and all the estimates were reported with 95% confidence intervals (95% CI).

**Table S1. Breakdown of intensive induction therapies**

| **Intensive induction Regimen** | **All**  **N=227 (%)** | **CL-**  **n=189 (%)** | **CL+**  **n=38 (%)** |
| --- | --- | --- | --- |
| R-EPOCH | 221 (97) | 186 (98.41) | 35 (92) |
| R-HyperCVAD | 1 (<1) | 0 (0) | 1 (3) |
| R-CODOX-M/R-IVAC | 2 (<1) | 1 (<1) | 1 (3) |
| Others | 3 (1) | 2 (1) | 1 (3) |
| MACOP-B | 1 | 1 | 0 |
| R maxi CHOP | 1 | 0 | 1 |
| R2CHOP | 1 | 1 | 0 |

Abbreviations: R-EPOCH: etoposide, prednisone, vincristine, cyclophosphamide, doxorubicin, and rituximab, R-Hyper-CVAD (rituximab, hyperfractionated cyclophosphamide, vincristine, doxorubicin, and dexamethasone with methotrexate and cytarabine), R-CODOX-M/R-IVAC (rituximab, cyclophosphamide, vincristine, doxorubicin, and high-dose methotrexate; rituximab, ifosfamide, etoposide, and high-dose cytarabine), MACOP-B: methotrexate with leucovorin rescue, doxorubicin, cyclophosphamide, vincristine, prednisone, and bleomycin, R2CHOP: lenalidomide plus R-CHOP.

First-line treatment regimens were divided into two groups: R-CHOP/ R-CHOP like (rituximab, cyclophosphamide, doxorubicin, vincristine, and prednisone) and intensive induction therapies. Intensive induction therapies included dose-adjusted R-EPOCH (etoposide, prednisone, vincristine, cyclophosphamide, doxorubicin, and rituximab), R-Hyper-CVAD (rituximab, hyperfractionated cyclophosphamide, vincristine, doxorubicin, and dexamethasone with methotrexate and cytarabine), R-CODOX-M/R-IVAC (rituximab, cyclophosphamide, vincristine, doxorubicin, and high-dose methotrexate; rituximab, ifosfamide, etoposide, and high-dose cytarabine), MACOP-B (methotrexate with leucovorin rescue, doxorubicin, cyclophosphamide, vincristine, prednisone, and bleomycin), and others. Detailed breakdown of the intensive induction therapies categorized by the two groups is shown above in **Table S1**.

**Table S2. Treatment Patterns and Causes of Death**

| **Variable** | **All**  **N= 588 (%)** | **CL-**  **n=503 (%)** | **CL+**  **n=85 (%)** | **p-value** |
| --- | --- | --- | --- | --- |
| Treatment |  |  |  | 0.26 |
| R-CHOP and R-CHOP like | 361 (61) | 314 (62) | 47 (55) |  |
| Intensive induction therapies | 227 (39) | 189 (38) | 38 (45) |  |
| Autologous HCT |  |  |  | **0.005** |
| No | 524 (89) | 456 (91) | 68 (80) |  |
| Yes | 63 (11) | 46 (9) | 17 (20) |  |
| CAR-T |  |  |  | 0.54 |
| No | 566 (96) | 485 (96) | 81 (95) |  |
| Yes | 22 (4) | 18 (4) | 4 (5) |  |
| Allogeneic HCT |  |  |  | 1.0 |
| No | 580 (99) | 496 (99) | 84 (99) |  |
| Yes | 8 (1) | 7 (1) | 1 (1) |  |
| Cause of death if deceased |  |  |  | 0.44 |
| Disease progression | 68 (34) | 54 (33) | 14 (36) |  |
| Organ failure | 64 (32) | 50 (31) | 14 (36) |  |
| Infection | 19 (9) | 18 (11) | 1 (2) |  |
| Other causes | 51 (25) | 41 (25) | 10 (26) |  |

Abbreviations: HCT= Hematopoietic cell transplantation, CAR-T= Chimeric antigen receptor T-cell therapy, CR=Complete response, PR=Partial response, SD=Stable disease, PD=Progressive disease

### Table S3. Response rates after first line treatment in CL+ and CL- patients.

| **Variable** | **All patients**  **N=588 (%)** | **CL-**  **n=503 (%)** | **CL+**  **n=85 (%)** |
| --- | --- | --- | --- |
| ORR | 505 (86) | 439 (87) | 66 (78) |
| CR | 427(73) | 377 (75) | 50 (59) |
| PR | 78 (13) | 62 (12) | 16 (19) |
| SD | 51 (8) | 43 (9) | 6 (7) |
| PD | 49 (6) | 21 (4) | 13 (15) |

### Abbreviations: ORR- objective response rate, CR – complete response, PR – partial response, SD – stable disease, PD – progressive disease

**Table S4. PFS Modeling (Univariable and Multivariable analysis*)**

| **Variable** | **Univariable** | | **Multivariable** | |
| --- | --- | --- | --- | --- |
|  | **HR (95% CI)** | **p-value** | **HR (95% CI)** | **p-value** |
| CL |  |  |  |  |
| CL- | Referent |  | 1.00 |  |
| CL+ | 2.15 (1.6,2.89) | **< 0.001** | 2.04 (1.47, 2.84) | **< 0.001** |
| Age at treatment (years) | 1 (0.99, 1.01) | 0.45 |  |  |
| Gender |  |  |  |  |
| Male | Referent |  |  |  |
| Female | 1.08 (0.86, 1.36) | 0.51 |  |  |
| Race/Ethnicity |  |  |  |  |
| White | Referent |  |  |  |
| Other races | 0.86 (0.51, 1.44) | 0.56 |  |  |
| ECOG PS |  |  |  |  |
| 0-1 | Referent |  |  |  |
| 2-3 | 0.98 (0.78, 1.24) | 0.90 |  |  |
| Stage |  |  |  |  |
| 1-2 | Referent |  |  |  |
| 3-4 | 1.01 (0.67, 1.54) | 0.95 |  |  |
| B-symptoms |  |  |  |  |
| No | Referent |  |  |  |
| Yes | 0.86 (0.67, 1.1) | 0.22 |  |  |
| Bulky Disease |  |  |  |  |
| No | Referent |  |  |  |
| Yes | 0.79 (0.62, 0.99) | **0.049** | 0.89 (0.69, 1.16) | 0.40 |
| Albumin low |  |  |  |  |
| No | Referent |  |  |  |
| Yes | 0.80 (0.64, 1.02) | 0.07 | 0.86 (0.67, 1.11) | 0.23 |
| LDH > ULN |  |  |  |  |
| No | Referent |  |  |  |
| Yes | 1.17 (0.89, 1.53) | 0.26 |  |  |
| Cell of origin |  |  |  |  |
| GCB | Referent |  |  |  |
| Non-GCB | 1 (0.77, 1.31) | 0.98 |  |  |
| Unknown | 0.96 (0.72, 1.28) | 0.67 |  |  |
| DHL/THL |  |  |  |  |
| No | Referent |  |  |  |
| Yes | 1.53 (1.04, 2.26) | **0.03** | 1.38 (0.92, 2.05) | 0.12 |
| R-IPI Prognostic score |  |  |  |  |
| 0 | Referent |  |  |  |
| 1-2 | 0.93 (0.46, 1.89) | 0.84 | 0.90 (0.44, 1.84) | 0.76 |
| 3-5 | 0.94 (0.45, 1.99) | 0.87 | 0.83 (0.39, 1.78) | 0.63 |
| Time to Treatment |  |  |  |  |
| 0-14 days | Referent |  |  |  |
| >15 days | 1.05 (0.77, 1.44) | 0.76 |  |  |
| Frontline Therapy |  |  |  |  |
| R-CHOP | Referent |  |  |  |
| Intensive Induction | 0.87 (0.69, 1.11) | 0.26 |  |  |

Abbreviations: HR, hazard ratio; CL-, circulating lymphoma cells absent; CL+, circulating lymphoma cells present; LDH, lactate dehydrogenase; ULN, upper limit of normal, GCB-Germinal Center B Cell like, DHL -Double Hit Lymphoma, THL-Triple Hit Lymphoma, R-IPI, Revised international prognostic index.

*Censored at 14 years

**Table S5. OS Modeling (Univariable and Multivariable analysis)**

| **Variable** | **Univariable** | | **Multivariable** | |
| --- | --- | --- | --- | --- |
|  | **HR (95% CI)** | **p-value** | **HR (95% CI)** | **p-value** |
| CL |  |  |  |  |
| CL- | Referent |  |  |  |
| CL+ | 1.72 (1.22, 2.44) | **0.002** | 1.61 (1.1, 2.36) | **0.01** |
| Age at treatment (years) | 1 (0.99, 1.01) | 0.86 |  |  |
| Gender |  |  |  |  |
| Male | Referent |  |  |  |
| Female | 0.98 (0.74, 1.29) | 0.87 |  |  |
| Race/Ethnicity |  |  |  |  |
| White | Referent |  |  |  |
| Other races | 0.65 (0.32, 1.32) | 0.23 |  |  |
| ECOG PS |  |  |  |  |
| 0-1 | Referent |  |  |  |
| 2-3 | 1.01 (0.76, 1.34) | 0.93 |  |  |
| Stage |  |  |  |  |
| 1-2 | Referent |  |  |  |
| 3-4 | 0.96 (0.58, 1.60) | 0.88 |  |  |
| B-symptoms |  |  |  |  |
| No | Referent |  |  |  |
| Yes | 0.82 (0.61, 1.11) | 0.18 |  |  |
| Bulky Disease |  |  |  |  |
| No | Referent |  |  |  |
| Yes | 0.77 (0.53, 0.95) | **0.02** | 0.77 (0.56, 1.06) | 0.11 |
| Albumin low |  |  |  |  |
| No | Referent |  |  |  |
| Yes | 0.87 (0.66, 1.16) | 0.35 |  |  |
| LDH >ULN |  |  |  |  |
| No | Referent |  |  |  |
| Yes | 1.01 (0.73-1.39) | 0.96 |  |  |
| Cell of origin |  |  |  |  |
| GCB | Referent |  |  |  |
| Non-GCB | 1.04 (0.75, 1.44) | 0.82 |  |  |
| Unknown | 0.82 (0.58, 1.17) | 0.27 |  |  |
| DHL/THL |  |  |  |  |
| No | Referent |  |  |  |
| Yes | 1.6 (1.02, 2.53) | **0.04** | 1.48 (0.93, 2.35 | 0.10 |
| R-IPI Prognostic score |  |  |  |  |
| 0 | Referent |  |  |  |
| 1-2 | 0.90 (0.37, 2.19) | 0.81 | 0.86 (0.35, 2.12) | 0.74 |
| 3-5 | 1.16 (0.46, 2.93) | 0.76 | 1.12 (0.43, 2.89) | 0.82 |
| Time to Treatment |  |  |  |  |
| 0-14 days | Referent |  |  |  |
| >15 days | 0.80 (0.55, 1.16) | 0.24 |  |  |
| Frontline Therapy |  |  |  |  |
| R-CHOP | Referent |  |  |  |
| Intensive Induction | 0.96 (0.72, 1.27) | 0.76 |  |  |

Abbreviations: HR, hazard ratio; CL-, circulating lymphoma cells absent; CL+, circulating lymphoma cells present; LDH, lactate dehydrogenase; ULN, upper limit of normal, GCB-Germinal Center B Cell like, DHL -Double Hit Lymphoma, THL-Triple Hit Lymphoma, R-IPI, Revised international prognostic index.

**Table S6. Baseline characteristics based on stage 1-3 CL-, stage 4 CL- and CL+ cohorts**

| **Variable** | ***Stage 1-3 CL-**  **n=193 (%)** | ***Stage 4 CL-**  **N=308 (%)** | **CL+**  **n=85 (%)** | **p-value** |
| --- | --- | --- | --- | --- |
| Median age, years (range) | 69 (22-90) | 71 (23-90) | 67 (39-97) | **0.05** |
| Gender |  |  |  | 0.26 |
| Male | 96 (50) | 157 (51) | 51 (60) |  |
| Female | 97 (50) | 151 (49) | 34 (40) |  |
| Race |  |  |  | 0.53 |
| White | 181 (94) | 287 (93) | 82 (97) |  |
| Other | 12 (6) | 21 (7) | 3 (3) |  |
| ECOG PS |  |  |  | 0.14 |
| 0-1 | 109 (57) | 182 (59) | 58 (70) |  |
| 2-3 | 81 (43) | 125 (41) | 25 (30) |  |
| B-symptoms |  |  |  | 0.67 |
| No | 81 (48) | 139 (52) | 44 (53) |  |
| Yes | 88 (52) | 130 (48) | 39 (47) |  |
| Bulky Disease |  |  |  | **< 0.001** |
| No | 106 (55) | 174 (57) | 71 (85) |  |
| Yes | 87 (45) | 132 (43) | 12 (15) |  |
| Low albumin |  |  |  | **< 0.001** |
| No | 104 (54) | 167 (54) | 66 (79) |  |
| Yes | 88 (46) | 141 (46) | 18 (21) |  |
| LDH >ULN |  |  |  | **0.02** |
| No | 58 (30) | 65 (21) | 28 (33) |  |
| Yes | 135 (70) | 243 (79) | 57 (67) |  |
| Cell of origin |  |  |  | 0.24 |
| GCB | 83 (43) | 121 (39) | 29 (34) |  |
| Non-GCB | 60 (31) | 98 (32) | 37 (44) |  |
| Unknown | 50 (26) | 89 (29) | 19 (22) |  |
| *MYC* rearrangement |  |  |  | **0.004** |
| No | 153 (87) | 245 (88) | 56 (74) |  |
| Yes | 22 (13) | 32 (12) | 20 (26) |  |
| DHL/THL |  |  |  | **0.004** |
| No | 160 (91) | 260 (94) | 63 (82) |  |
| Yes | 15 (9) | 17 (6) | 14 (18) |  |
| R-IPI Prognostic score |  |  |  | **< 0.001** |
| 0 | 16 (8) | 0 | 0 |  |
| 1-2 | 171 (89) | 219 (71) | 72 (85) |  |
| 3-5 | 6 (3) | 89 (29) | 13 (15) |  |

### Abbreviations: CL-, circulating lymphoma cells absent; CL+, circulating lymphoma cells present; LDH, lactate dehydrogenase; ULN, upper limit of normal, GCB-Germinal Center B Cell like, DHL -Double Hit Lymphoma, THL-Triple Hit Lymphoma, R-IPI, Revised international prognostic index.

*Total n=501 in CL- group due to the missing stage in two patients

### Table S7. Response rates after first line treatment in stage 1-3 CL-, stage 4 CL- and CL+ cohorts

| **Variable** | ***Stage 1-3 CL-**  **N=193 (%)** | ***Stage 4 CL-**  **N=308 (%)** | **CL+**  **N=85 (%)** |
| --- | --- | --- | --- |
| ORR | 165 (85) | 272 (88) | 66 (78) |
| CR | 145 (75) | 230 (75) | 50 (59) |
| PR | 20 (10) | 42 (14) | 16 (19) |
| SD | 22 (11) | 21 (7) | 6 (7) |
| PD | 6 (3) | 15 (5) | 13 (15) |

### Abbreviations: ORR- objective response rate, CR – complete response, PR – partial response, SD – stable disease, PD – progressive disease

*Total n=501 in CL- group due to the missing stage in two patients

**Figure S1. Consort Diagram**


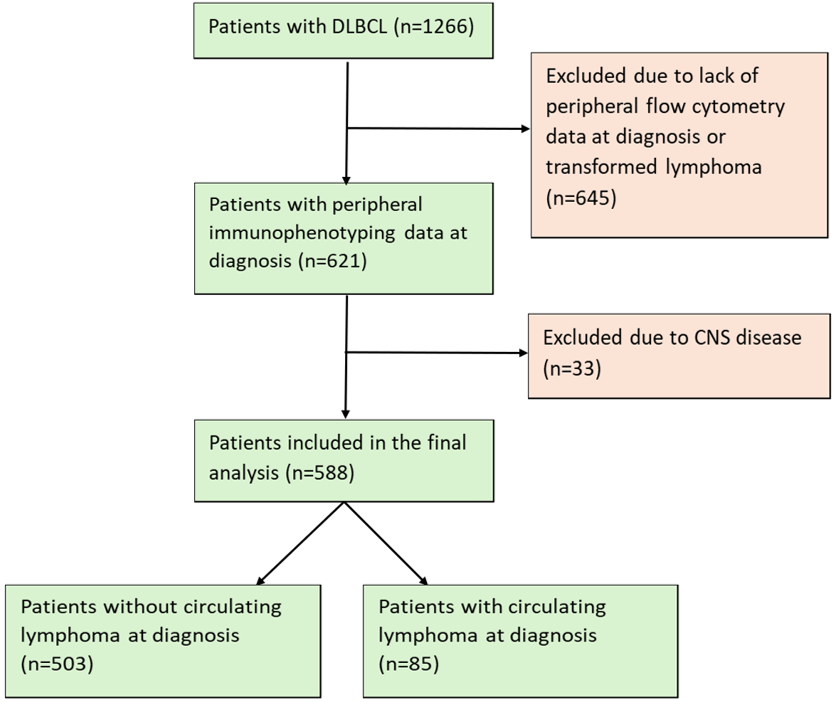


**Figure S2. Progression-free survival between CL+ and CL- groups A) among R-CHOP treated patients B) among intensive induction therapy**

**A)**

**
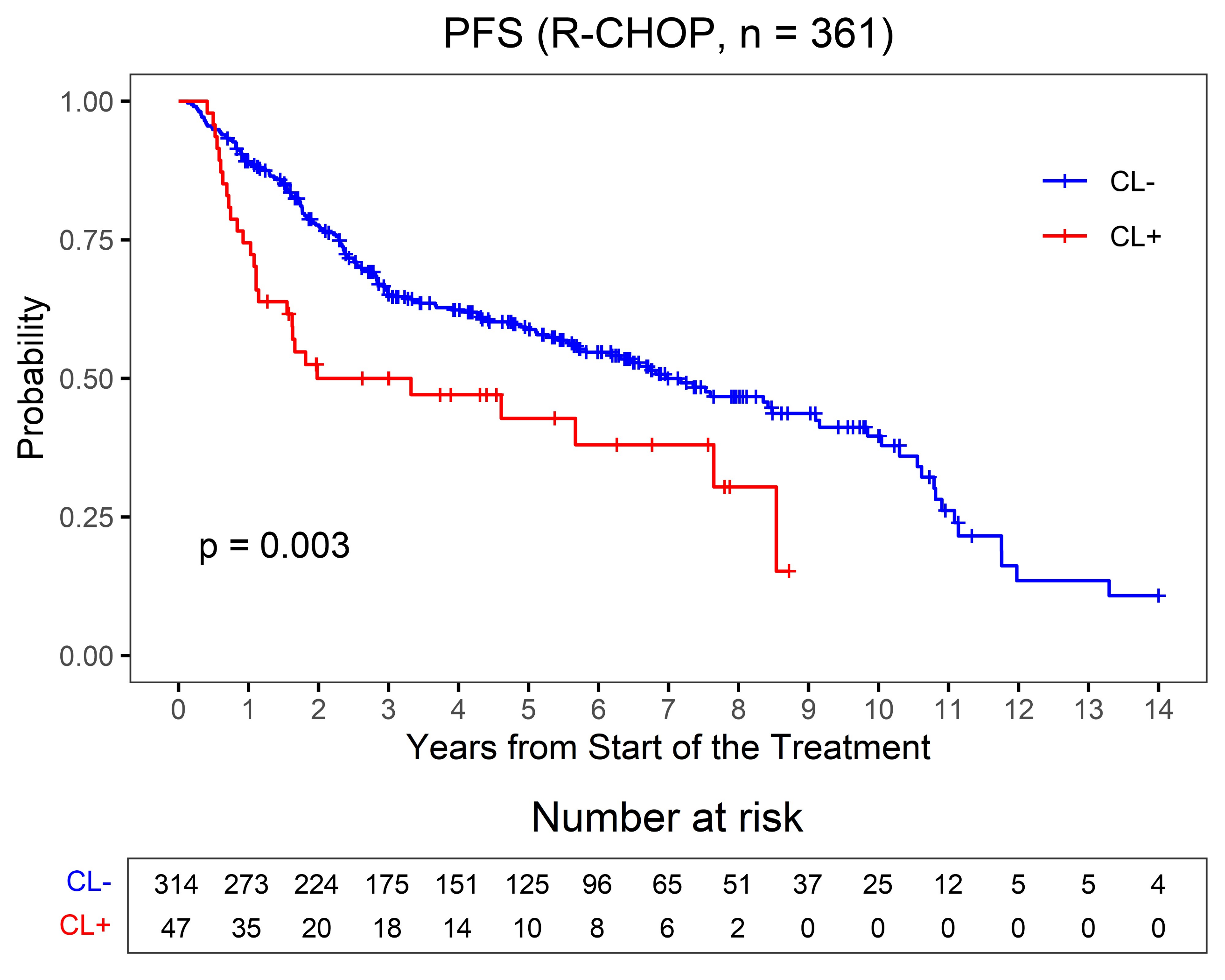
**

**B)**


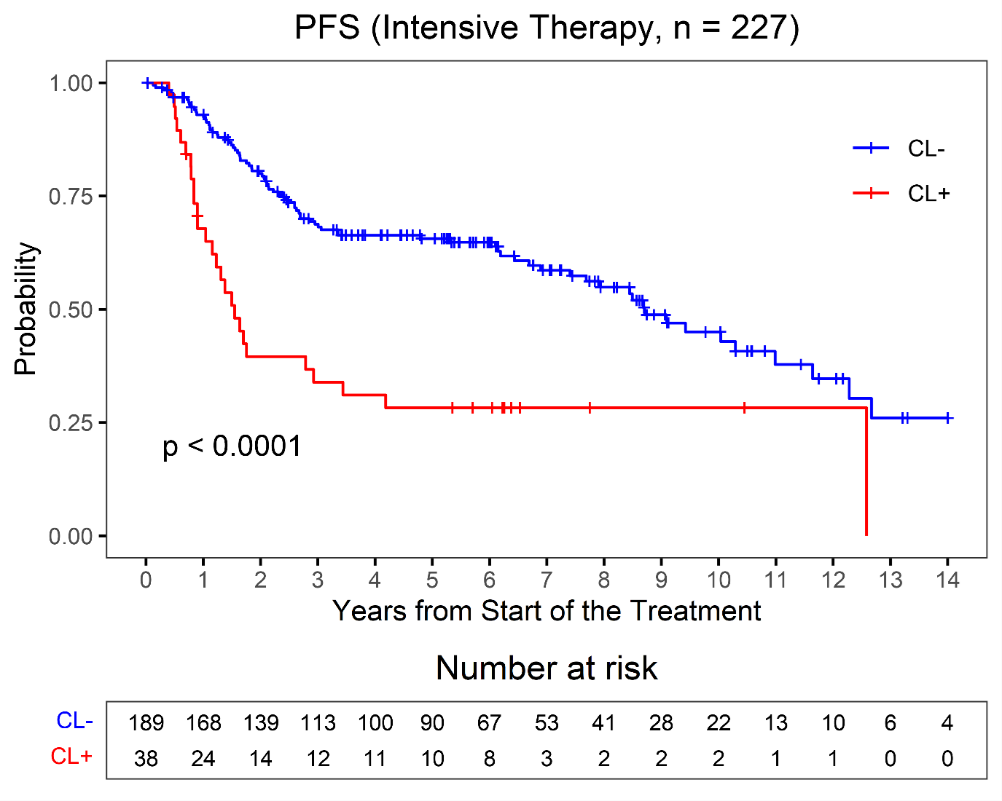


**Figure S3. Overall survival between CL+ and CL- groups A) among intensive induction therapy B) among R-CHOP treated patients**

**A)**


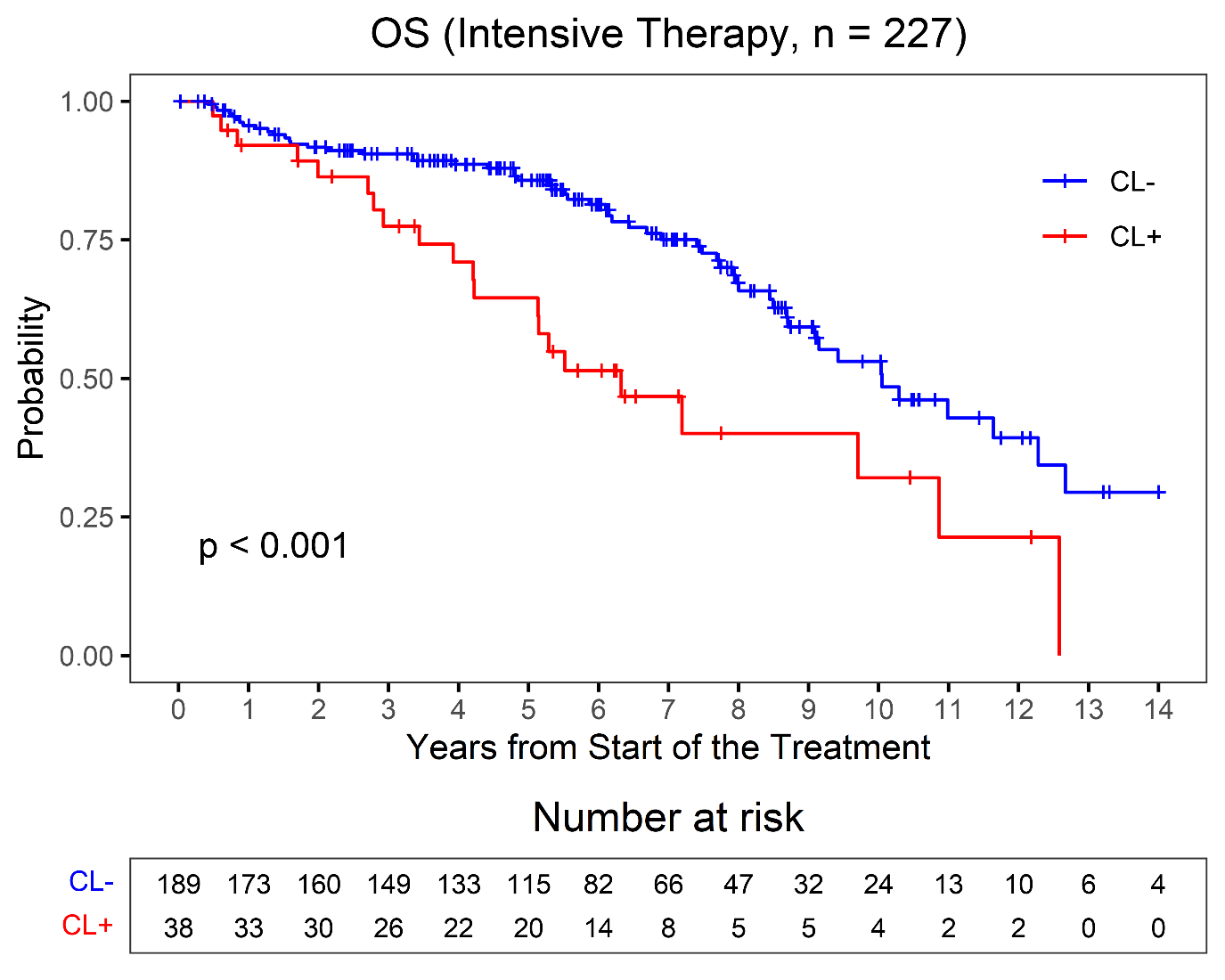


**B)**

**
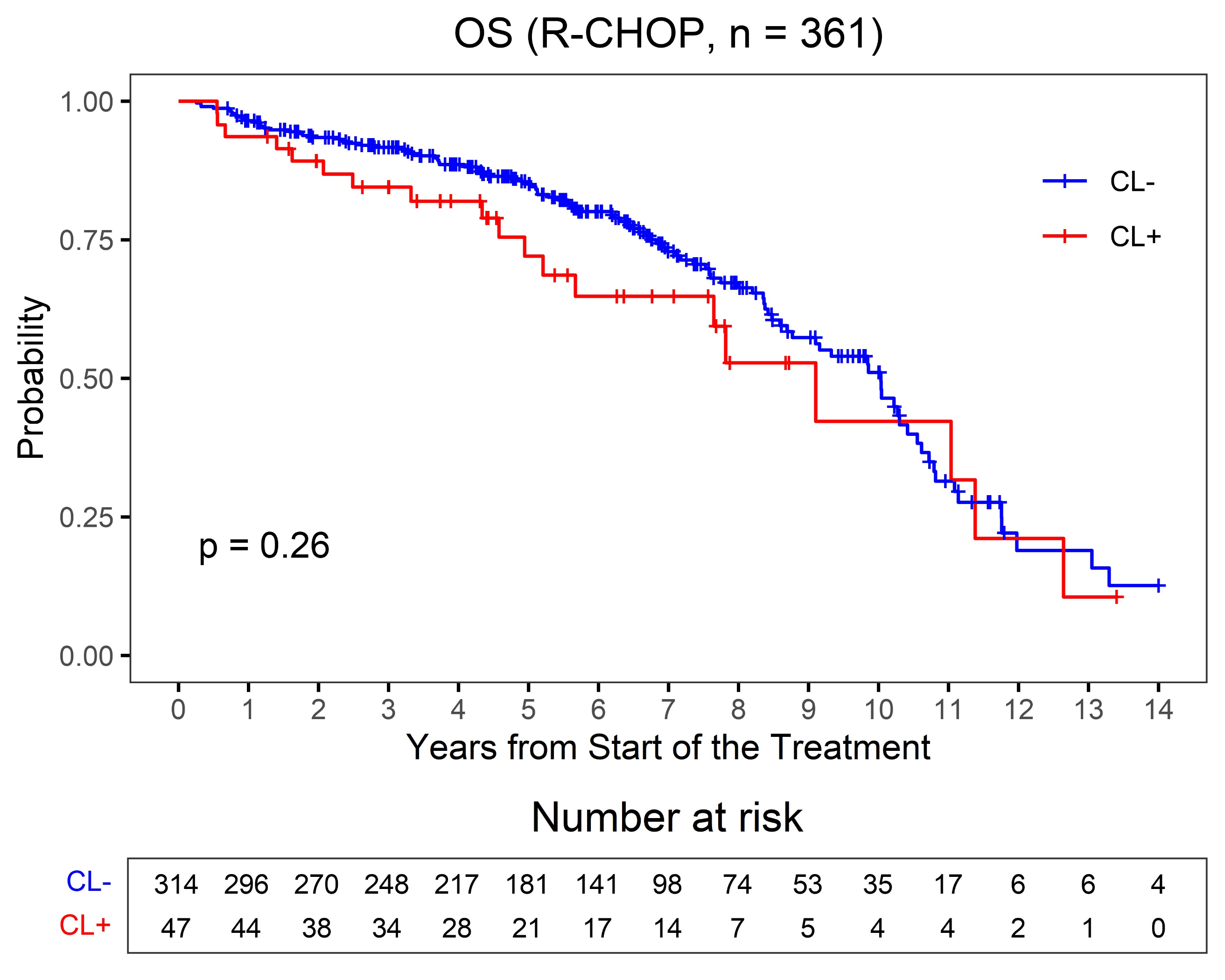
**

**Figure S4. Survival outcomes between stage 1-3 CL-, stage 4 CL- and CL+ groups A) Progression-free survival B) Overall survival**

**A)**


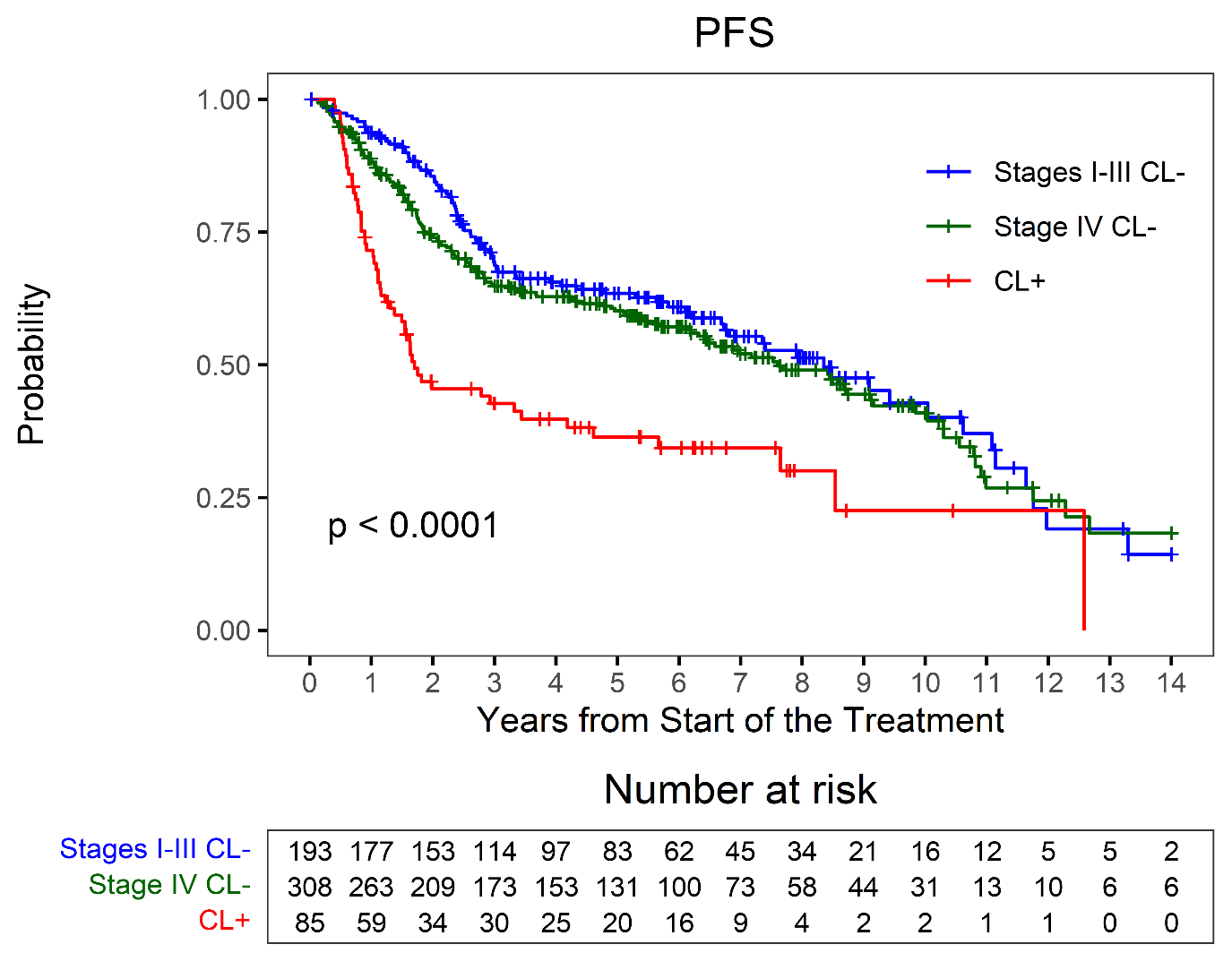


**B)**


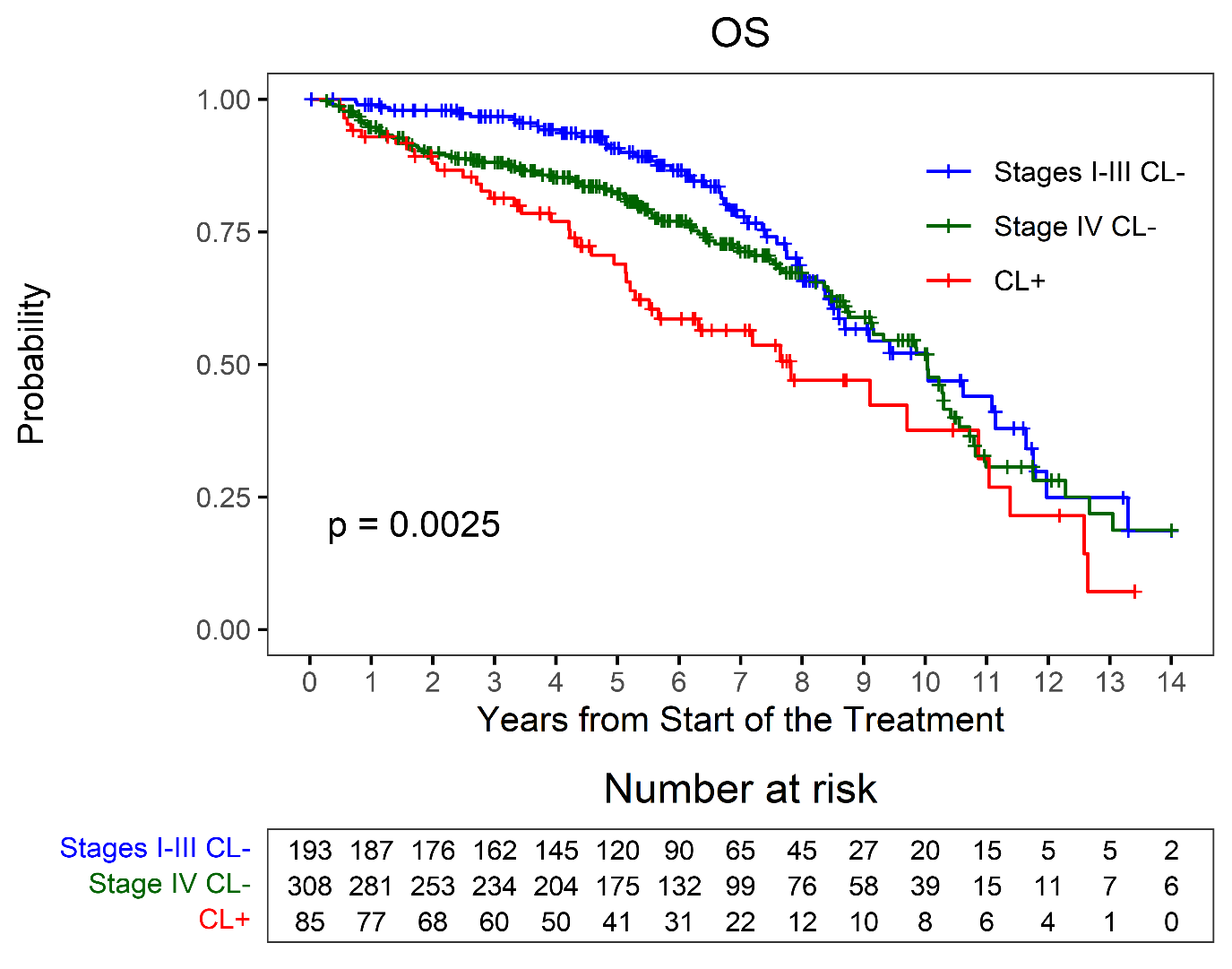

Supplement: Supplementary file 1 — Supplementary Material 1 [file 13045_2024_1658_MOESM1_ESM.docx]
